# Guanidinium dioxidobis(picolinato-κ^2^ *N*,*O*)(picolinato-κ*O*)uranate(VI)

**DOI:** 10.1107/S1600536812035465

**Published:** 2012-09-05

**Authors:** Vitalii I. Mishkevich, Mikhail S. Grigoriev, Alexandre M. Fedosseev, Philippe Moisy

**Affiliations:** a A.N. Frumkin Institute of Physical Chemistry and Electrochemistry, Russian Academy of Sciences, 31 Leninsky Prospekt, 119071 Moscow, Russian Federation; b Comissariat a l’Energy Atomique (CEA), Marcoule, DEN/DRCP, BP 17171 30207 Bagnols-sur-Ceze, France

## Abstract

In the title compound, (CH_6_N_3_)[U(C_6_H_4_NO_2_)_3_O_2_], the uranyl group is coordinated by two O and two N atoms from two chelating picolinate ligands, and one O atom from a third picolinate ligand. The coordination environment of the U^VI^ atom (N_2_O_5_) is distorted penta­gonal–bipyramidal. In the crystal, all amino groups are involved in the formation of N—H⋯O and N—H⋯N hydrogen bonds, which link cations and anions into layers parallel to the *ac* plane.

## Related literature

For the disordered crystal structure of a related complex without guanidinium in which the uranyl ion is chelated by two picolinato ligands and coordinated *via* the O atom of a picolinic acid mol­ecule, see: Grechishnikova *et al.* (2007 ▶).
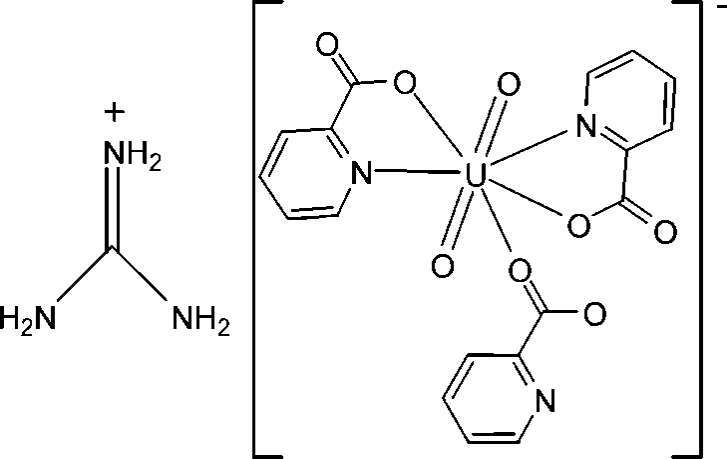


## Experimental

### 

#### Crystal data

(CH_6_N_3_)[U(C_6_H_4_NO_2_)_3_O_2_]
*M* *_r_* = 696.42
Orthorhombic, 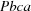

*a* = 16.3842 (4) Å
*b* = 13.1678 (3) Å
*c* = 21.2743 (4) Å
*V* = 4589.80 (18) Å^3^
*Z* = 8
Mo *K*α radiation
μ = 7.13 mm^−1^
*T* = 293 K
0.18 × 0.06 × 0.04 mm

#### Data collection

Bruker Kappa APEXII CCD diffractometer
Absorption correction: multi-scan (*SADABS*; Sheldrick, 2007 ▶) *T*_min_ = 0.360, *T*_max_ = 0.764
77357 measured reflections
6604 independent reflections
3818 reflections with *I* > 2σ(*I*)
*R*_int_ = 0.093

#### Refinement

*R*[*F* ^2^ > 2σ(*F* ^2^)] = 0.035
*wR*(*F* ^2^) = 0.075
*S* = 1.01
6604 reflections
307 parameters
H-atom parameters constrained
Δρ_max_ = 1.07 e Å^−3^
Δρ_min_ = −0.71 e Å^−3^

### 

Data collection: *APEX2* (Bruker, 2006 ▶); cell refinement: *SAINT-Plus* (Bruker, 1998 ▶); data reduction: *SAINT-Plus*; program(s) used to solve structure: *SHELXS97* (Sheldrick, 2008 ▶); program(s) used to refine structure: *SHELXL97* (Sheldrick, 2008 ▶); molecular graphics: *SHELXTL* (Sheldrick, 2008 ▶); software used to prepare material for publication: *SHELXTL*.

## Supplementary Material

Crystal structure: contains datablock(s) I, global. DOI: 10.1107/S1600536812035465/cv5297sup1.cif

Structure factors: contains datablock(s) I. DOI: 10.1107/S1600536812035465/cv5297Isup2.hkl

Additional supplementary materials:  crystallographic information; 3D view; checkCIF report

## Figures and Tables

**Table 1:** Hydrogen-bond geometry (Å, °)

*D*—H⋯*A*	*D*—H	H⋯*A*	*D*⋯*A*	*D*—H⋯*A*
N11—H11*A*⋯O4^i^	0.86	2.02	2.859 (6)	166
N11—H11*B*⋯O6^ii^	0.86	2.12	2.918 (6)	154
N12—H12*A*⋯O3^i^	0.86	2.18	3.033 (6)	169
N12—H12*B*⋯N3	0.86	2.24	3.042 (7)	156
N13—H13*B*⋯O8	0.86	2.10	2.847 (6)	146
N13—H13*C*⋯O6^ii^	0.86	2.35	3.083 (6)	144

Symmetry codes: (i) 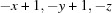; (ii) 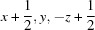.

**Fig. 1.**
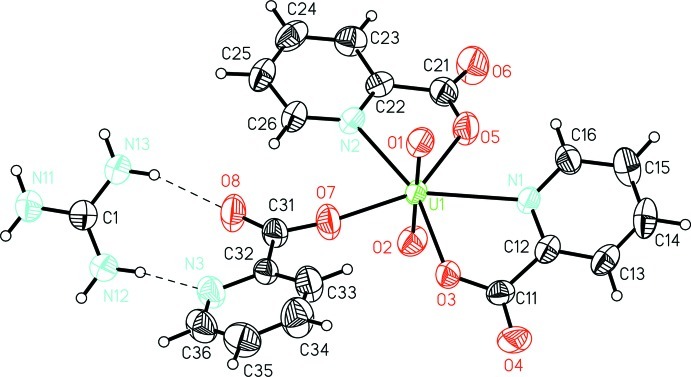
A view of the title compound showing the atom-labelling scheme. Displacement ellipsoids are drawn at the 50% probability level and H atoms are represented by circles of arbitrary size. Dashed lines indicate the hydrogen bonds.

**Fig. 2.**
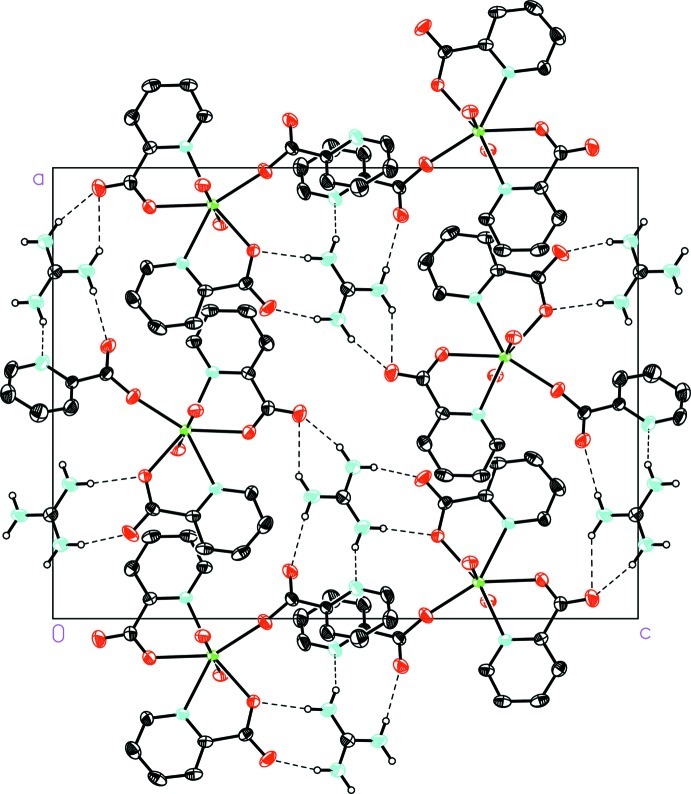
A portion of the crystal packing. Displacement ellipsoids are drawn at the 30% probability level and H atoms are represented by circles of arbitrary size. H atoms of anions omitted for clarity. Dashed lines indicate the hydrogen bonds.

## supplementary crystallographic information

### Comment

The title structure contains complex anions, in which dioxidocations UO_2_^2+^ are surrounded by two bidentate-chelating picolinate anions, coordinated by N and O atoms with the formation of 5-membered cycles, and one monodentate picolinate anion, coordinated by an O atom of the carboxylic group (Fig. 1). The guanidinium cation is located in the outer sphere. The UO_2_ groups are almost linear and symmetric. Coordination polyhedra of U atoms are distorted pentagonal bipyramids. The main distortions of coordination polyhedra are the differences between O—U—O and O—U—N angles in the equatorial plane. The U—O distances for O atoms of monodentate picolinate ligands are shorter, compared to U—O distances for bidentate ligands. The U—N distances are longer than U—O ones. Guanidinium cations act as proton donors for 6 H-bonds (2 bonds from each amino group) (Table 1) with O atoms of carboxylic groups and N atoms of organic anions. Each cation is connected to three complex anions forming layers parallel to the (010) plane (Fig. 2). This compound is the first anionic picolinate complex of uranyl and the first example of monodentate coordination of picolinate anion to an actinide cation.

### Experimental

The solid UO_3_.H_2_O was dissolved in 0.5 *M* aqueous solution of picolinic acid at 1:2 molar ratio. Then an equimolar quantity of guanidinium picolinate solution was added. This solution had been prepared by the neutralization of 1 *M* aqueous solution of guanidinium carbonate by an equimolar quantity of solid picolinic acid.

Light-yellow crystals were obtained by heating the reaction mixture up to 120 °C in a sealed glass tube.

### Refinement

The H atoms were placed in calculated positions with displacement parameters constrained to 1.2 times the U_iso_ of their parent atoms.

The largest electron density peak on the final difference Fourier-synthesis is 1.066 e Å^-3^ (0.93 Å from U1), the deepest hole is -0.707 e Å^-3^ (1.16 Å from O7).

### Crystal data

**Table:**

(CH_6_N_3_)[U(C_6_H_4_NO_2_)_3_O_2_]	*F*(000) = 2640
*M**_r_* = 696.42	*D*_x_ = 2.016 Mg m^−^^3^
Orthorhombic, *P**b**c**a*	Mo *K*α radiation, λ = 0.71073 Å
Hall symbol: -P 2ac 2ab	Cell parameters from 9491 reflections
*a* = 16.3842 (4) Å	θ = 6.2–24.9°
*b* = 13.1678 (3) Å	µ = 7.13 mm^−^^1^
*c* = 21.2743 (4) Å	*T* = 293 K
*V* = 4589.80 (18) Å^3^	Fragment, light-yellow
*Z* = 8	0.18 × 0.06 × 0.04 mm

### Data collection

**Table:**

Bruker Kappa APEXII CCD diffractometer	6604 independent reflections
Radiation source: fine-focus sealed tube	3818 reflections with *I* > 2σ(*I*)
Graphite monochromator	*R*_int_ = 0.093
ω and φ scans	θ_max_ = 30.0°, θ_min_ = 4.1°
Absorption correction: multi-scan (*SADABS*; Sheldrick, 2007)	*h* = −23→23
*T*_min_ = 0.360, *T*_max_ = 0.764	*k* = −18→18
77357 measured reflections	*l* = −27→29

### Refinement

**Table:**

Refinement on *F*^2^	Primary atom site location: structure-invariant direct methods
Least-squares matrix: full	Secondary atom site location: difference Fourier map
*R*[*F*^2^ > 2σ(*F*^2^)] = 0.035	Hydrogen site location: inferred from neighbouring sites
*wR*(*F*^2^) = 0.075	H-atom parameters constrained
*S* = 1.01	*w* = 1/[σ^2^(*F*_o_^2^) + (0.0317*P*)^2^] where *P* = (*F*_o_^2^ + 2*F*_c_^2^)/3
6604 reflections	(Δ/σ)_max_ = 0.001
307 parameters	Δρ_max_ = 1.07 e Å^−^^3^
0 restraints	Δρ_min_ = −0.71 e Å^−^^3^

### Special details

**Table:**

Geometry. All e.s.d.'s (except the e.s.d. in the dihedral angle between two l.s. planes) are estimated using the full covariance matrix. The cell e.s.d.'s are taken into account individually in the estimation of e.s.d.'s in distances, angles and torsion angles; correlations between e.s.d.'s in cell parameters are only used when they are defined by crystal symmetry. An approximate (isotropic) treatment of cell e.s.d.'s is used for estimating e.s.d.'s involving l.s. planes.
Refinement. Refinement of *F*^2^ against ALL reflections. The weighted *R*-factor *wR* and goodness of fit *S* are based on *F*^2^, conventional *R*-factors *R* are based on *F*, with *F* set to zero for negative *F*^2^. The threshold expression of *F*^2^ > σ(*F*^2^) is used only for calculating *R*-factors(gt) *etc*. and is not relevant to the choice of reflections for refinement. *R*-factors based on *F*^2^ are statistically about twice as large as those based on *F*, and *R*- factors based on ALL data will be even larger.

### Fractional atomic coordinates and isotropic or equivalent isotropic displacement parameters (Å^2^)

**Table:**

	*x*	*y*	*z*	*U*_iso_*/*U*_eq_	
U1	0.418415 (10)	0.592900 (13)	0.227356 (7)	0.03351 (6)	
O1	0.4611 (2)	0.7128 (3)	0.24392 (16)	0.0495 (9)	
O2	0.3762 (2)	0.4717 (3)	0.21274 (15)	0.0496 (9)	
O3	0.3164 (2)	0.6513 (3)	0.15747 (14)	0.0466 (9)	
O4	0.1888 (2)	0.6949 (3)	0.13249 (18)	0.0660 (12)	
O5	0.4135 (2)	0.5504 (3)	0.33518 (15)	0.0560 (10)	
O6	0.4548 (3)	0.4710 (4)	0.42111 (17)	0.0715 (13)	
O7	0.4928 (2)	0.5876 (3)	0.13743 (17)	0.0580 (10)	
O8	0.6048 (2)	0.5233 (4)	0.09453 (17)	0.0659 (12)	
N1	0.2885 (2)	0.6762 (3)	0.27861 (17)	0.0365 (9)	
N2	0.5468 (2)	0.5021 (3)	0.27319 (18)	0.0381 (9)	
N3	0.5676 (3)	0.6053 (3)	−0.0183 (2)	0.0532 (12)	
C11	0.2433 (3)	0.6807 (3)	0.1704 (2)	0.0399 (12)	
C12	0.2272 (3)	0.7013 (3)	0.2395 (2)	0.0366 (11)	
C13	0.1551 (3)	0.7450 (4)	0.2593 (3)	0.0471 (13)	
H13A	0.1138	0.7596	0.2307	0.057*	
C14	0.1456 (4)	0.7664 (4)	0.3215 (3)	0.0584 (15)	
H14A	0.0976	0.7958	0.3360	0.070*	
C15	0.2082 (4)	0.7438 (5)	0.3626 (3)	0.0621 (16)	
H15A	0.2032	0.7585	0.4052	0.075*	
C16	0.2786 (3)	0.6989 (4)	0.3393 (2)	0.0479 (13)	
H16A	0.3207	0.6839	0.3672	0.057*	
C21	0.4669 (3)	0.4998 (4)	0.3676 (2)	0.0474 (13)	
C22	0.5445 (3)	0.4778 (4)	0.3340 (2)	0.0391 (11)	
C23	0.6114 (4)	0.4354 (4)	0.3651 (3)	0.0503 (14)	
H23A	0.6084	0.4192	0.4076	0.060*	
C24	0.6818 (3)	0.4182 (4)	0.3318 (3)	0.0548 (15)	
H24A	0.7279	0.3929	0.3519	0.066*	
C25	0.6833 (3)	0.4389 (4)	0.2682 (3)	0.0493 (13)	
H25A	0.7295	0.4251	0.2443	0.059*	
C26	0.6142 (3)	0.4808 (4)	0.2410 (3)	0.0459 (13)	
H26A	0.6151	0.4946	0.1981	0.055*	
C31	0.5434 (3)	0.5758 (4)	0.0917 (2)	0.0445 (13)	
C32	0.5242 (3)	0.6342 (4)	0.0327 (2)	0.0389 (11)	
C33	0.4686 (4)	0.7118 (5)	0.0309 (3)	0.0601 (16)	
H33A	0.4392	0.7287	0.0668	0.072*	
C34	0.4562 (4)	0.7646 (5)	−0.0237 (3)	0.0735 (19)	
H34A	0.4200	0.8188	−0.0250	0.088*	
C35	0.4985 (4)	0.7357 (5)	−0.0765 (3)	0.0681 (18)	
H35A	0.4908	0.7688	−0.1146	0.082*	
C36	0.5523 (4)	0.6569 (5)	−0.0712 (2)	0.0592 (16)	
H36A	0.5805	0.6376	−0.1072	0.071*	
N11	0.8357 (3)	0.4011 (4)	−0.0134 (2)	0.0632 (14)	
H11A	0.8357	0.3657	−0.0474	0.076*	
H11B	0.8791	0.4044	0.0093	0.076*	
N12	0.7021 (3)	0.4469 (4)	−0.0293 (2)	0.0658 (14)	
H12A	0.7003	0.4122	−0.0635	0.079*	
H12B	0.6596	0.4796	−0.0169	0.079*	
N13	0.7708 (3)	0.5039 (4)	0.0568 (2)	0.0719 (16)	
H13B	0.7278	0.5362	0.0688	0.086*	
H13C	0.8146	0.5066	0.0790	0.086*	
C1	0.7694 (3)	0.4500 (4)	0.0039 (3)	0.0514 (14)	

### Atomic displacement parameters (Å^2^)

**Table:**

	*U*^11^	*U*^22^	*U*^33^	*U*^12^	*U*^13^	*U*^23^
U1	0.02556 (9)	0.04300 (10)	0.03197 (8)	0.00403 (9)	0.00182 (8)	0.00058 (8)
O1	0.039 (2)	0.054 (2)	0.056 (2)	0.0013 (19)	0.0014 (16)	−0.0038 (18)
O2	0.042 (2)	0.047 (2)	0.059 (2)	0.0028 (18)	−0.0024 (17)	0.0004 (17)
O3	0.038 (2)	0.065 (2)	0.0370 (18)	0.0144 (19)	−0.0010 (15)	−0.0033 (16)
O4	0.055 (3)	0.082 (3)	0.060 (2)	0.029 (2)	−0.028 (2)	−0.015 (2)
O5	0.045 (2)	0.082 (3)	0.0405 (19)	0.016 (2)	0.0066 (17)	0.0084 (18)
O6	0.067 (3)	0.104 (4)	0.043 (2)	0.015 (3)	0.008 (2)	0.026 (2)
O7	0.053 (2)	0.077 (3)	0.0440 (19)	0.014 (2)	0.0162 (18)	0.0023 (19)
O8	0.050 (3)	0.096 (3)	0.051 (2)	0.029 (2)	0.0112 (19)	0.008 (2)
N1	0.028 (2)	0.041 (2)	0.040 (2)	0.0035 (17)	0.0023 (18)	0.0013 (18)
N2	0.029 (2)	0.044 (2)	0.042 (2)	0.0005 (18)	0.000 (2)	0.0056 (19)
N3	0.054 (3)	0.060 (3)	0.045 (2)	−0.007 (2)	0.012 (2)	−0.003 (2)
C11	0.038 (3)	0.036 (3)	0.046 (3)	0.001 (2)	−0.008 (2)	−0.005 (2)
C12	0.030 (3)	0.028 (2)	0.051 (3)	0.000 (2)	0.002 (2)	0.002 (2)
C13	0.032 (3)	0.037 (3)	0.072 (4)	0.003 (2)	−0.003 (3)	0.001 (2)
C14	0.049 (4)	0.048 (3)	0.079 (4)	0.012 (3)	0.024 (3)	0.000 (3)
C15	0.069 (4)	0.064 (4)	0.054 (3)	0.016 (3)	0.018 (3)	0.001 (3)
C16	0.048 (3)	0.058 (4)	0.038 (3)	0.008 (3)	0.004 (2)	−0.004 (2)
C21	0.048 (3)	0.056 (4)	0.038 (3)	0.003 (3)	0.001 (2)	0.002 (2)
C22	0.036 (3)	0.038 (3)	0.043 (3)	−0.001 (2)	−0.005 (2)	0.001 (2)
C23	0.055 (4)	0.045 (3)	0.051 (3)	0.006 (3)	−0.008 (3)	0.006 (2)
C24	0.038 (3)	0.047 (3)	0.079 (4)	0.009 (3)	−0.013 (3)	0.000 (3)
C25	0.033 (3)	0.045 (3)	0.070 (4)	0.006 (2)	0.005 (3)	0.001 (3)
C26	0.034 (3)	0.048 (3)	0.055 (3)	0.006 (3)	0.003 (2)	0.002 (2)
C31	0.042 (3)	0.052 (4)	0.040 (3)	−0.004 (3)	0.009 (2)	−0.002 (2)
C32	0.035 (3)	0.045 (3)	0.036 (3)	−0.005 (2)	0.005 (2)	−0.004 (2)
C33	0.062 (4)	0.069 (4)	0.049 (3)	0.015 (3)	0.011 (3)	0.001 (3)
C34	0.075 (5)	0.071 (5)	0.074 (4)	0.025 (4)	0.006 (4)	0.006 (4)
C35	0.081 (5)	0.073 (5)	0.050 (4)	0.000 (4)	−0.002 (3)	0.012 (3)
C36	0.067 (4)	0.070 (4)	0.041 (3)	−0.014 (4)	0.015 (3)	0.003 (3)
N11	0.048 (3)	0.089 (4)	0.053 (3)	0.017 (3)	0.000 (2)	−0.012 (3)
N12	0.045 (3)	0.093 (4)	0.059 (3)	0.012 (3)	−0.007 (2)	−0.030 (3)
N13	0.039 (3)	0.111 (4)	0.065 (3)	0.018 (3)	−0.004 (2)	−0.033 (3)
C1	0.045 (4)	0.062 (4)	0.048 (3)	−0.001 (3)	0.003 (3)	−0.005 (3)

### Geometric parameters (Å, º)

**Table:**

U1—O1	1.762 (4)	C21—C22	1.486 (7)
U1—O2	1.766 (4)	C22—C23	1.397 (7)
U1—O3	2.366 (3)	C23—C24	1.371 (8)
U1—O5	2.362 (3)	C23—H23A	0.9300
U1—O7	2.269 (3)	C24—C25	1.381 (8)
U1—N1	2.631 (4)	C24—H24A	0.9300
U1—N2	2.609 (4)	C25—C26	1.386 (7)
O3—C11	1.288 (5)	C25—H25A	0.9300
O4—C11	1.218 (5)	C26—H26A	0.9300
O5—C21	1.298 (6)	C31—C32	1.506 (7)
O6—C21	1.217 (6)	C32—C33	1.371 (7)
O7—C31	1.287 (6)	C33—C34	1.369 (8)
O8—C31	1.221 (6)	C33—H33A	0.9300
N1—C16	1.335 (6)	C34—C35	1.372 (9)
N1—C12	1.345 (6)	C34—H34A	0.9300
N2—C26	1.330 (6)	C35—C36	1.365 (9)
N2—C22	1.334 (6)	C35—H35A	0.9300
N3—C36	1.340 (7)	C36—H36A	0.9300
N3—C32	1.351 (6)	N11—C1	1.316 (7)
C11—C12	1.518 (7)	N11—H11A	0.8600
C12—C13	1.379 (7)	N11—H11B	0.8600
C13—C14	1.360 (8)	N12—C1	1.310 (7)
C13—H13A	0.9300	N12—H12A	0.8600
C14—C15	1.381 (8)	N12—H12B	0.8600
C14—H14A	0.9300	N13—C1	1.331 (7)
C15—C16	1.389 (7)	N13—H13B	0.8600
C15—H15A	0.9300	N13—H13C	0.8600
C16—H16A	0.9300		
			
O1—U1—O2	178.53 (16)	O6—C21—O5	123.2 (5)
O1—U1—O7	89.03 (15)	O6—C21—C22	121.9 (5)
O2—U1—O7	91.94 (15)	O5—C21—C22	114.9 (4)
O1—U1—O5	91.81 (15)	N2—C22—C23	122.1 (5)
O2—U1—O5	86.77 (15)	N2—C22—C21	116.4 (4)
O7—U1—O5	146.05 (13)	C23—C22—C21	121.5 (5)
O1—U1—O3	96.59 (15)	C24—C23—C22	118.8 (5)
O2—U1—O3	84.64 (14)	C24—C23—H23A	120.6
O7—U1—O3	81.95 (12)	C22—C23—H23A	120.6
O5—U1—O3	131.53 (12)	C23—C24—C25	119.3 (5)
O1—U1—N2	90.92 (14)	C23—C24—H24A	120.4
O2—U1—N2	88.11 (14)	C25—C24—H24A	120.4
O7—U1—N2	82.41 (13)	C24—C25—C26	118.2 (5)
O5—U1—N2	63.64 (12)	C24—C25—H25A	120.9
O3—U1—N2	162.51 (12)	C26—C25—H25A	120.9
O1—U1—N1	82.23 (14)	N2—C26—C25	123.1 (5)
O2—U1—N1	97.65 (14)	N2—C26—H26A	118.4
O7—U1—N1	142.81 (12)	C25—C26—H26A	118.4
O5—U1—N1	70.66 (12)	O8—C31—O7	124.1 (5)
O3—U1—N1	63.45 (12)	O8—C31—C32	120.1 (5)
N2—U1—N1	133.52 (12)	O7—C31—C32	115.7 (5)
C11—O3—U1	128.3 (3)	N3—C32—C33	122.6 (5)
C21—O5—U1	127.9 (3)	N3—C32—C31	114.6 (5)
C31—O7—U1	170.5 (4)	C33—C32—C31	122.9 (5)
C16—N1—C12	116.9 (4)	C34—C33—C32	120.0 (5)
C16—N1—U1	126.3 (3)	C34—C33—H33A	120.0
C12—N1—U1	116.8 (3)	C32—C33—H33A	120.0
C26—N2—C22	118.2 (4)	C33—C34—C35	118.6 (6)
C26—N2—U1	125.0 (3)	C33—C34—H34A	120.7
C22—N2—U1	116.7 (3)	C35—C34—H34A	120.7
C36—N3—C32	115.6 (5)	C36—C35—C34	117.9 (6)
O4—C11—O3	125.8 (5)	C36—C35—H35A	121.0
O4—C11—C12	119.1 (5)	C34—C35—H35A	121.0
O3—C11—C12	115.0 (4)	N3—C36—C35	125.2 (5)
N1—C12—C13	123.6 (5)	N3—C36—H36A	117.4
N1—C12—C11	115.1 (4)	C35—C36—H36A	117.4
C13—C12—C11	121.3 (5)	C1—N11—H11A	120.0
C14—C13—C12	118.8 (5)	C1—N11—H11B	120.0
C14—C13—H13A	120.6	H11A—N11—H11B	120.0
C12—C13—H13A	120.6	C1—N12—H12A	120.0
C13—C14—C15	119.1 (5)	C1—N12—H12B	120.0
C13—C14—H14A	120.4	H12A—N12—H12B	120.0
C15—C14—H14A	120.4	C1—N13—H13B	120.0
C14—C15—C16	118.8 (5)	C1—N13—H13C	120.0
C14—C15—H15A	120.6	H13B—N13—H13C	120.0
C16—C15—H15A	120.6	N12—C1—N11	121.9 (5)
N1—C16—C15	122.8 (5)	N12—C1—N13	119.1 (5)
N1—C16—H16A	118.6	N11—C1—N13	118.9 (5)
C15—C16—H16A	118.6		

### Hydrogen-bond geometry (Å, º)

**Table:**

*D*—H···*A*	*D*—H	H···*A*	*D*···*A*	*D*—H···*A*
N11—H11*A*···O4^i^	0.86	2.02	2.859 (6)	166
N11—H11*B*···O6^ii^	0.86	2.12	2.918 (6)	154
N12—H12*A*···O3^i^	0.86	2.18	3.033 (6)	169
N12—H12*B*···N3	0.86	2.24	3.042 (7)	156
N13—H13*B*···O8	0.86	2.10	2.847 (6)	146
N13—H13*C*···O6^ii^	0.86	2.35	3.083 (6)	144

Symmetry codes: (i) −*x*+1, −*y*+1, −*z*; (ii) *x*+1/2, *y*, −*z*+1/2.

## References

[bb1] Bruker (1998). *SAINT-Plus* Bruker AXS Inc., Madison, Wisconsin, USA.

[bb2] Bruker (2006). *APEX2* Bruker AXS Inc., Madison, Wisconsin, USA.

[bb3] Grechishnikova, E. V., Peresypkina, E. V., Virovets, A. V., Mikhailov, Yu. N. & Serezhkina, L. B. (2007). *Russ. J. Coord. Chem.* **33**, 458–465.

[bb4] Sheldrick, G. M. (2007). *SADABS.* Bruker AXS Inc., Madison, Wisconsin, USA.

[bb5] Sheldrick, G. M. (2008). *Acta Cryst.* A**64**, 112–122. 10.1107/S0108767307043930 18156677

